# Low-Temperature Reduction Synthesis of γ–Fe_2_O_3−x_@biochar Catalysts and Their Combining with Peroxymonosulfate for Quinclorac Degradation

**DOI:** 10.3390/ijerph192416790

**Published:** 2022-12-14

**Authors:** Mei-e Zhong, Gongsong Tong, Jingchun Sun, Nan Zhou, Chunxia Ding, Xiangying Liu, Austin Merchant, Xuguo Zhou

**Affiliations:** 1 School of Chemistry and Materials Science, Hunan Agricultural University, No.1 Nongda Road, Furong District, Changsha 410128, China; 2 College of Plant Protection, Hunan Agricultural University, No.1 Nongda Road, Furong District, Changsha 410128, China; 3 Hunan Provincial Key Laboratory for Biology and Control of Weeds, Hunan Agricultural University, No.1 Nongda Road, Furong District, Changsha 410125, China; 4 Department of Entomology, University of Kentucky, S-225 Agricultural Science Center North, Lexington, KY 40546-0091, USA

**Keywords:** low–temperature reduction, γ–Fe_2_O_3_, biochar, peroxymonosulfate, quinclorac

## Abstract

Biochar loading mixed–phase iron oxide shows great advantages as a promising catalyst owing to its eco–friendliness and low cost. Here, γ–Fe_2_O_3−x_@biochar (E/Fe–N–BC) composite was successfully prepared by the sol–gel method combined with low–temperature (280 °C) reduction. The Scanning Electron Microscope (SEM) result indicated that γ–Fe_2_O_3−x_ particles with the size of approximately 200 nm were well–dispersed on the surface of biochar. The CO derived from biomass pyrolysis is the main reducing component for the generation of Fe (II). The high content of Fe (II) contributed to the excellent catalytic performance of E/Fe–N–BC for quinclorac (QNC) degradation in the presence of peroxymonosulfate (PMS). The removal efficiency of 10 mg/L of QNC was 100% within 30 min using 0.3 g/L γ–Fe_2_O_3−x_@biochar catalyst and 0.8 mM PMS. The radical quenching experiments and electron paramagnetic resonance analysis confirmed that •OH and SO_4_•^−^ were the main radicals during the degradation of QNC. The facile and easily mass–production of γ–Fe_2_O_3−x_@biochar with high catalytic activity make it a promising catalyst to activate PMS for the removal of organic pollutants.

## 1. Introduction

Currently, carboxylic acid herbicides are widely applied in controlling the growth of certain grassy weeds, such as barnyard grass, moleplant seed, and crab grass in paddies [1,2]. Quinclorac (QNC), an excellent synthetic auxin selective herbicide, has been adopted for controlling barnyard grasses in rice fields. However, attenuation of quinclorac in soil is very slow due to its stable molecular structure [3,4,5]. It is reported that QNC has harmful effects on sensitive crops [6] and aquatic animals [7]. Hence, it is essential to develop highly efficient and feasible strategies for removal of QNC in the environment.

In recent years, persulfate–involved advanced oxidation processes (AOPs) have shown great superiority over the remediation of recalcitrant organic pollutants due to their high efficiency [8,9,10] and wide pH range [11]. Generally, persulfate, such as peroxymonosulfate (PMS) and peroxydisulfate (PDS), can produce reactive oxygen species (ROS) (including SO_4_•^−^ and •OH) by breaking the O–O bond. However, due to the strong binding energy of the peroxide (O–O) bond in persulfate, the formation of active species in AOPs is closely dependent on the catalytic ability of the catalyst. Compared with PDS, PMS is more easily activated due to its asymmetric structure. Therefore, numerous approaches have been employed to activate PMS, including physical (microwave [12], heat [13], UV irradiation [14], and ultrasound [15]) and chemical (alkali [16], transition metal ions, metal–based catalysts [17], and carbon–based materials) activation. Among them, iron–based materials [18], such as Fe (Ⅲ) catalysts (e.g., FeOOH, γ–Fe_2_O_3_, and α–Fe_2_O_3_) have attracted widespread attention due to their non–toxicity, eco–friendliness, high mineral efficiency, low energy demand, and facile synthesis. Unfortunately, most of these catalysts show low catalytic activities to PMS because the electron transfer from PMS (HSO_5_^−^) to Fe (III) during the reaction process is very slow (Equation (1)). Then, the generated Fe (II) can serve as active species for cleaving PMS to produce sulfate radicals (${\mathrm{SO}}_{4}{•}^{-}$) (Equation (2)) [18,19].

(1) $${\mathrm{HSO}}_{5}{}^{-}+{\mathrm{Fe}}^{3+}\to {\mathrm{Fe}}^{2+}+{\mathrm{SO}}_{5}{•}^{-}+H^{+}\;$$

(2) $${\mathrm{HSO}}_{5}{}^{-}+{\mathrm{Fe}}^{2+}\to {\mathrm{Fe}}^{3+}+{\mathrm{SO}}_{4}{•}^{-}+{\mathrm{OH}}^{-}$$

It has been reported that introducing heterogeneous metals to form mixed metals composites, such as CuFe_2_O_4_ [20,21], CoFe_2_O_4_ [22,23,24], CuO–γ–Fe_2_O_3_ [25], and γ–Fe_2_O_3_/CeO_2_ [26], are favorable for accelerating the electron transfer from PMS to Fe (III). Although these strategies can enhance the catalytic activity of Fe (III) catalysts, the biotoxicity of heterogeneous metals such as Co, Cu, and Ce impedes their field application. Therefore, the assembly of environmentally friendly mixed–phase iron oxides (e.g., α–Fe_2_O_3_@Fe_3_O_4_, Fe^0^/Fe_3_O_4_) may be a promising strategy to increase the catalytic activity of Fe (III) catalysts [27]. Unfortunately, the synthesis of mixed–phase iron oxides was usually carried out under high temperature (>500 °C) or in the presence of strong reductants, such as CO, H_2_, or plant extraction [28,29,30]. In addition, it is necessary to load mixed–phase iron oxides with a solid matrix (e.g., biochar, graphene, and montmorillonite) to increase their active sites by providing good distribution. Therefore, the synchronous low–temperature (<500 °C) synthesis and solid–phase loading of mixed–phase iron oxide in a reasonable way may exhibit great economic and cost advantages. 

Wasted biomass, one type of natural renewable resources, has been widely used as a reductant to yield reductive substances for reducing manganese ore and iron ore. Moreover, during pyrolysis, biomass can serve as a carbon resource to generate biochar for loading catalysts [31]. Hence, an Fe (III) compound/biomass mixture would be an appropriate precursor for the synthesis of mixed–phase iron oxides–biochar composite with superior catalytic performance. Moreover, some research has shown that the reduction behavior of iron ores (hematite) was controlled by the surface reaction of particles [32]. For example, Pang et al. [33] found that the reaction temperature decreased by 80 °C after the particle size of iron ores dropped to 6.5 μm from 107.5 μm. Furthermore, the sol–gel method has been widely used to prepare a number of metal nanoclusters [34]. Then, nanosized particles can dramatically accelerate the surface reaction, which is likely to reduce the synthesis temperature. 

Herein, we (1) employed ethylenediamine tetra–acetic acid ferric sodium salt (FeNaEDTA), a Fe–containing chelating agent as an iron precursor. Sesame husks, a typical kind of agricultural leftover material, was used as a reductant and carbon resource for the synthesis of γ–Fe_2_O_3−x_@biochar (E/Fe–N–BC) composites through the sol–gel method combined with low–temperature (280 °C) reduction reaction; (2) characterized the structure and morphology of E/Fe–N–BC by X–ray diffraction (XRD), N_2_ adsorption/desorption technique, scanning electron microscopy (SEM), field emission transmission electron microscopy (TEM), and X–ray photoelectron spectroscopy (XPS); (3) determined the factors (QNC initial concentration, PMS dosage, E/Fe–N–BC dosage, pH, co–existing anions, and humic acid (HA)) on the removal efficiency of QNC; and (4) identified the active species and proposed the degradation mechanism of QNC in E/Fe–N–BC/PMS system. 

## 2. Experimental Sections

### 2.1. Reagents and Materials

Sesame husks were obtained from local farmers (Ningxiang county, Hunan province). Ethylenediaminetetraacetic acid sodium ferric salt (FeNaEDTA, C_10_H_12_FeNaN_2_O_8_, AR), and peroxymonosulfate (PMS, KHSO_5_·0.5KHSO_4_·0.5K_2_SO_4_, AR) were purchased from Aladdin (Shanghai, China). Ethylene diamine tetra–acetic acid (C_10_H_16_N_2_O_8_, EDTA, AR), sulfuric acid (H_2_SO_4_, AR), sodium hydroxide (NaOH, AR), sodium dihydrogen phosphate (NaH_2_PO_4_, AR), sodium nitrate (NaNO_3_, AR), sodium chloride (NaCl, AR), and sodium bicarbonate (NaHCO_3_, AR) were purchased from Sinopharm (Beijing, China). Simulated pollutants, including quinclorac (QNC, ≥98%), dicamba (DM, ≥98%), 2, 4–dichlorophenoxyacetic acid (2,4–D, ≥98%), and atrazine (ATZ, ≥97%) were obtained from local pesticide companies without further purification. The water used in the experiment was deionized water (18.25 MΩ·cm). 

### 2.2. Preparation of Catalysts

The E/Fe–N–BC in this experiment were prepared by a sol–gel method combined with a low–temperature carbon thermal reduction method. Firstly, 1 g of sesame husks, 0.35 g of EDTA, and 0.5 g of FeNaEDTA were mixed with 50 mL of deionized water in a 300 mL porcelain crucible and stirred well to dissolve completely. Subsequently, the mixture underwent a sol–gel process at 60 °C in an oven for 5 h. Finally, the mixture in the porcelain crucible with the corresponding lid was transferred into a muffle furnace and heated at 280 °C for 4 h at a heating rate of 5 °C/min. After cooling to room temperature, the obtained material was labeled as E/Fe–N–BC. For comparison, the samples were prepared by the identical method but without EDTA, FeNaEDTA, or both, and they were denoted by Fe–N–BC, N–BC, or BC, respectively. The samples using FeNaEDTA and water as raw materials were prepared by the same method and named Fe–N–C.

### 2.3. Batch Experimental Processes

All degradation experiments were performed in 250 mL conical flasks. Typically, 100 mL of a certain concentration of quinclorac (QNC) was poured into a conical flask. Subsequently, PMS and as–obtained catalysts were added into the solution to initiate the reaction. Then, the mixture was transferred into an orbital shaking incubator with a shaking rate of 150 rpm at 25 °C and maintained for 30 min. At pre–defined time intervals, 1 mL of the reaction solution was taken out by a 10 mL polyethylene syringe and filtered with a 0.22 µm polyether sulfone membrane and added into a bottle containing 0.2 mL ethanol solution to stop the reaction. The effects of initial QNC concentrations (5~40 mg·L^−1^), humic acid (HA, 2~6 mg·L^−1^), and anions (HCO_3_^−^, NO_3_^−^, H_2_PO_4_^−^, Cl^−^, 5 mM) on QNC removal were also studied. The initial pH of reaction solution was adjusted with 0.1 M H_2_SO_4_ and 0.1 M NaOH solutions. Unless otherwise stated, pH of the reaction system was not adjusted. All experiments were conducted in parallel for three replicates, and the average value is presented. 

### 2.4. Characterization and Analytical Methods

The crystal structures of the materials were characterized by an X–ray diffractometer (XRD–6000, Shimadzu, Kyoto, Japan) at a scanning rate of 4° min^−1^ from 10° to 80°. The surface morphologies of the materials were observed with a transmission electron microscopy (FEI Tecnai G2 F20, Portland, OR, USA) and a scanning electron microscopy (TESCAN MIRA4 LMH, Shenzhen, China) equipped with an energy–dispersive X–ray spectroscopy (SEM–EDS, Ultim Max 40, Oxford, England). Magnetic hysteresis loops were recorded by a vibrating sample magnetometer (VSM, Quantum Design PPMS–9, San Diego, CA, USA) at room temperature. The specific surface areas (SSA), pore volume, and pore size distribution of materials were measured by N_2_ adsorption/desorption at 77K (BET, Kubo–X1000, Peod, Beijing, China). The functional groups of the samples were determined by an ALPHA flourier transform infrared (FT–IR) spectrometer (AXS–ALPHA, BRUKER, Karlsruhe, Germany) with a scan range of 400–4000 cm^−1^. The XPS study analysis was performed on a K–Alpha spectroscopy (US, Thermo Fisher Scientific, Waltham, MA, USA). The electron paramagnetic resonance (EPR) test was carried out on a JEOLJES–FA200 analyzer (Tokyo, Japan) for the detection of active species, where 5,5–dimethyl–1–pyrroline N–oxide (DMPO) was employed as a free radical capture agent. Fe leaching during the degradation process was determined by an atomic absorption spectrophotometer (TAS–990). Zeta potential at different pH values was measured by a Zetasizer Nano–Zs Instrument (ZEN3600 Malvern, Malvern, UK). The detailed measurement information of PMS residue concentration and electrochemical impedance spectroscopy (EIS) are shown in the Texts S2 and S3 of supporting information. 

The concentration of QNC was determined by High Performance Liquid Chromatography (HPLC, Agilent 1260 Infinity Ⅱ, California, USA) with an Agilent C_18_ column (5 µm, 250 mm × 46 mm). The total organic carbon (TOC) test was conducted by Shimadzu TOC–L CPN. The UV–detector wavelength was set as 240 nm, acetum (1 wt%)/methanol = 40:60 (*v*/*v*) was used as the mobile phase with a flow rate of 1 mL·min^−1^, and the column temperature was set at 30 °C. The reaction rates in all experiments were evaluated by pseudo–first order kinetic model, as show in Text S4. 

## 3. Results and Discussion

### 3.1. Characterization of Catalysts

**Figure 1:**
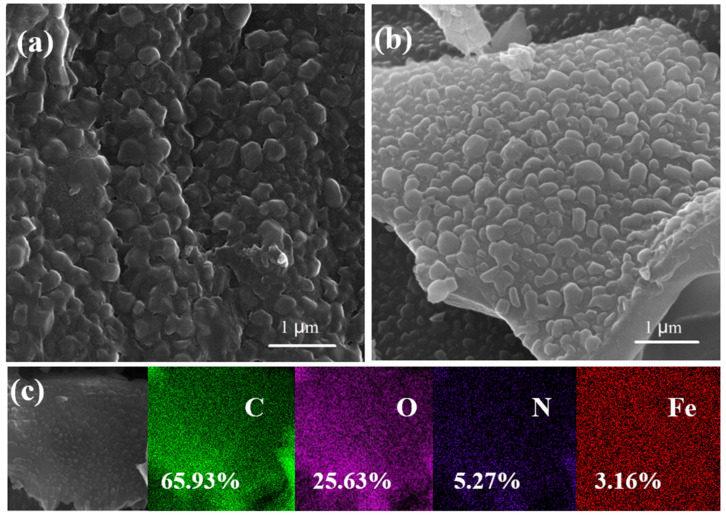
The SEM images of Fe–N–BC (**a**), E/Fe–N–BC (**b**), and the element mapping of E/Fe–N–BC (**c**).

The typical morphologies of BC, N–BC, Fe–N–BC, and E/Fe–N–BC were measured by scanning electron microscopy (SEM). The biochar (BC) (Figure S1a,b) derived from the sesame husks showed folded and sponge–like morphologies with tubular structures, indicating that low–temperature pyrolysis can retain their original structures. It is well–known that the tubular structure is beneficial for the dispersion and loading of catalysts. For N–BC (Figure S1c), it shows a morphology similar to BC after the addition of EDTA. Spherical aggregate particles with average particle size approximately 250 nm were observed on the surface of biochar in Fe–N–BC (Figure 1a), indicating that mixed iron oxides were formed on the surface of biochar. Compared with Fe–N–BC, the spherical–like particles adhering to the surface of E/Fe–N–BC exhibited higher dispersion with smaller size (~200 nm) (Figure 1b), which can be further confirmed by the statistical analysis diagrams of particle sizes in Fe–N–BC and E/Fe–N–BC (Figure S2). This is because the EDTA can act as a complexing agent to anchor Fe (III) ions in the sol–gel process [35], facilitating the formation of nanosized mixed iron oxides during the carbothermal reduction. Moreover, the EDS mapping images (Figure 1c) of E/Fe–N–BC confirm that C, N, O, and Fe species were homogeneously distributed on the surface of biochar. 

**Figure 2:**
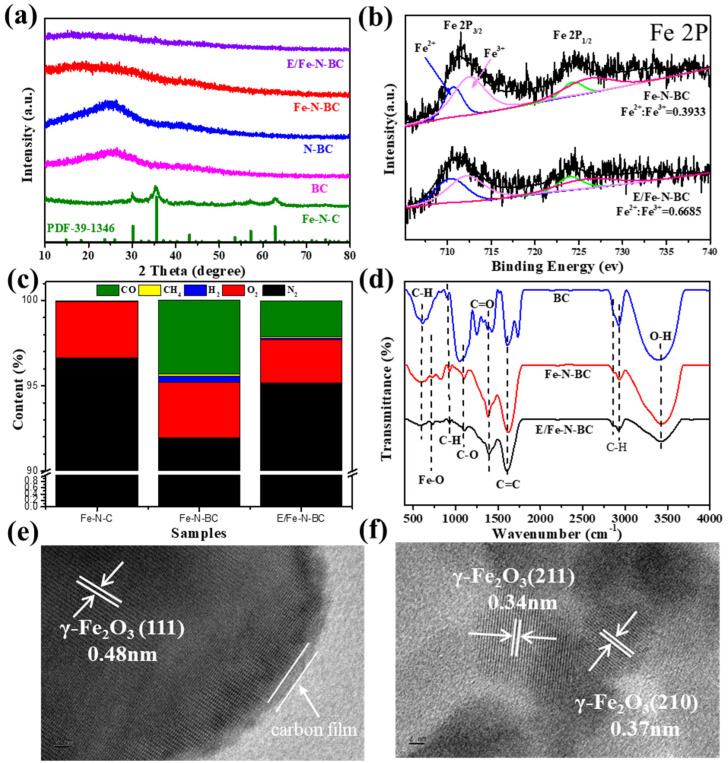
The XRD pattern of BC, N–BC, Fe–N–BC, E/Fe–N–BC, and Fe–N–C (**a**). The XPS spectra of Fe 2P, Fe–N–BC, and E/Fe–N–BC (**b**). The gas component during the synthesis of Fe–N–C, Fe–N–BC, and E/Fe–N–BC (**c**). The FT–IR of Fe–N–BC and E/Fe–N–BC (**d**). The TEM images of E/Fe–N–BC (**e**,**f**).

The XRD results (Figure 2a) show that BC and N–BC had a single peak at 25°, which can be assigned to the disorder carbon [36]. When FeNaEDTA alone was used as the precursor, the peaks at 30.24°, 35.63°, 43.28°, 53.73°, 57.21°, and 62.93° in Fe–N–C corresponded to (220), (311), (400), (422), (511), and (440) planes of γ–Fe_2_O_3_ (PDF–39–1346), respectively [37,38], indicating the formation of γ–Fe_2_O_3_. However, after the addition of biomass and EDTA, no crystalline peak could be observed on Fe–N–BC and E/Fe–N–BC, which may be attributed to the coating of deposition carbon.

To explore the transformation of iron species during pyrolysis, XPS analyses were adopted to investigate the surface elements contents and their chemical valence state of Fe–N–BC and E/Fe–N–BC. Their full survey spectra and elements contents are shown in Figure S3 and Table S2. It can be seen that the C and Fe contents in Fe–N–BC were 83.74% and 0.56%, respectively, indicating the successful loading of Fe on biochar. When EDTA was introduced during the synthesis, the C content (85.37%) in E/Fe–N–BC slightly enlarged with a gradual reduction of Fe (0.45%). This may have been caused by the formation of C from EDTA during pyrolysis. As shown in Figure S4, two obvious peaks located at 711.2 eV and 724.8 eV are assigned to Fe 2P_3/2_ and Fe 2P_1/2_, respectively. Additionally, two satellite signals at 718.8 eV and 732.6 eV are attributed to the typical characteristics of γ–Fe_2_O_3_ [25,39], which is consistent with the XRD results. This indicated that the iron species were presented mainly in the Fe (III) state for Fe–N–C. As shown in Figure 2b, due to the appearance of Fe (II) [25,37], the signal peaks of Fe 2P_3/2_ and Fe 2P_1/2_ for Fe–N–BC shifted to lower binding energies (711.0 eV and 724.6 eV). Moreover, a similar phenomenon was observed on E/Fe–N–BC. These results indicat that a portion of Fe (III) ions in Fe–N–BC and E/Fe–N–BC were converted into Fe (II) to generate mixed γ–Fe_2_O_3−x_ under low–temperature (280 °C) pyrolysis, which may be ascribed to the fact that FeNaEDTA, as an organic compound, can generate nanoscale Fe (III) precursors through a sol–gel reaction to decrease the reduction temperature. It is found that the proportions of Fe (II)/Fe (III) for Fe–N–BC and E/Fe–N–BC were calculated to be 0.3933 and 0.6685, respectively. High content Fe(II) contributed to the activation of PMS for the degradation of QNC. Moreover, the conversion of Fe(III) into Fe(II) could also be confirmed from the results of the magnetic hysteresis loop (Figure S5). It can be seen that the Ms values of Fe/N–C, Fe/N–BC, and E/Fe/N–BC were 15.19, 0.83, and 0.51 emu/g, respectively. Because the magnetization was derived only from γ–Fe_2_O_3_, the decreases of Ms in Fe/N–BC and E/Fe/N–BC may have been caused by the reduction of Fe content and the generation of Fe(II).

To investigate the reason for the reduction of Fe(III) into Fe(II) during pyrolysis, gas products during reaction were collected by using N_2_ as carrier gas, and the detailed acquisition procedure is described in Text S1. According to previous reports, CO and H_2_ can effectively reduce trivalence hematite into mixed–valence magnetite [40]. Moreover, because CH_4_ is the main pyrolysis gas from biomass decomposition, O_2_ exists largely in air atmosphere. Hence, the gas components of CO, CH_4_, H_2_, and O_2_ were measured by GC/MS. As depicted in Figure 2c, N_2_ as a carrier gas, accounted for the majority of gas components. Additionally, the volume fraction of CO was augmented from 0.05% to 4.30% in Fe–N–BC compared with Fe–N–C. This indicates that the pyrolysis of biomass (sesame shell) could produce reductive CO gas, which would be used as a reductant to conversion Fe (III) into Fe (II) for the formation of mixed γ–Fe_2_O_3−x_. In contrast, the yield of CO (2.12%) decreased in E/Fe–N–BC. This can be ascribed to the fact that EDTA, a strong complex agent for Fe (III) ions [35], would further decrease the particle size of Fe(III) precursors in E/Fe–N–BC by sol–gel reaction [33], which could accelerate the surface reduction reaction of iron oxides during the pyrolysis process. Consequently, a portion of CO was consumed, and more Fe (III) compounds were reduced and transformed into γ–Fe_2_O_3−x_ with higher Fe (II) content. 

The FT–IR spectra (Figure 2d) of 3430 cm^−1^, 2928 cm^−1^, 1621 cm^−1^, 1383 cm^−1^, 1100 cm^−1^, 727 cm^−1^, and 610 cm^−1^ corresponded to the vibration and stretching of O–H, C–H, C=C, C=O, C–O, O–Fe, and C–H, respectively [41]. The presence of the O–Fe peak in E/Fe–N–BC, and Fe–N–BC indicated the loading of iron oxides on the surface of biochar. 

The microstructure of E/Fe–N–BC was measured by TEM (Figure S6), in which iron oxides particles were well–coated with carbon film. Moreover, it can be seen from TEM (Figure 2e,f) that clear lattice fringes of 0.48, 0.37, and 0.34 nm corresponded to the (111), (210), and (211) planes γ–Fe_2_O_3_, respectively [42]. This further confirms the existence of γ–Fe_2_O_3_ in E/Fe–N–BC. Furthermore, an amorphous carbon film of approximately 2 nm was observed on the surface of γ–Fe_2_O_3_ particles. This result provides proof for the reason that no diffraction peak was detected on the XRD pattern. The results of TEM and XPS suggested that the γ–Fe_2_O_3−x_@biochar composite was obtained in E/Fe–N–BC by the sol–gel process combined with low temperature pyrolysis. 

As shown in Figure S7, all the materials exhibited a type Ⅲ adsorption curve with low porosity. The detailed information can be seen from Table S3, in which BC showed a small specific surface area of 3.6458 m^2^/g, with an average pore size of 6.23 nm. This may be attributed to the presence of a large number of tubular structures in biochar (Figure S1b), which can provide suitable sites for the loading of iron oxides. After the addition of iron salts, the specific surface areas of Fe–N–BC and E/Fe–N–BC obviously decreased, indicating that iron oxides were successfully loaded on biochar.

### 3.2. Catalytic PMS for the Removal of QNC

#### 3.2.1. Evaluation for the Catalytic Activity

**Figure 3:**
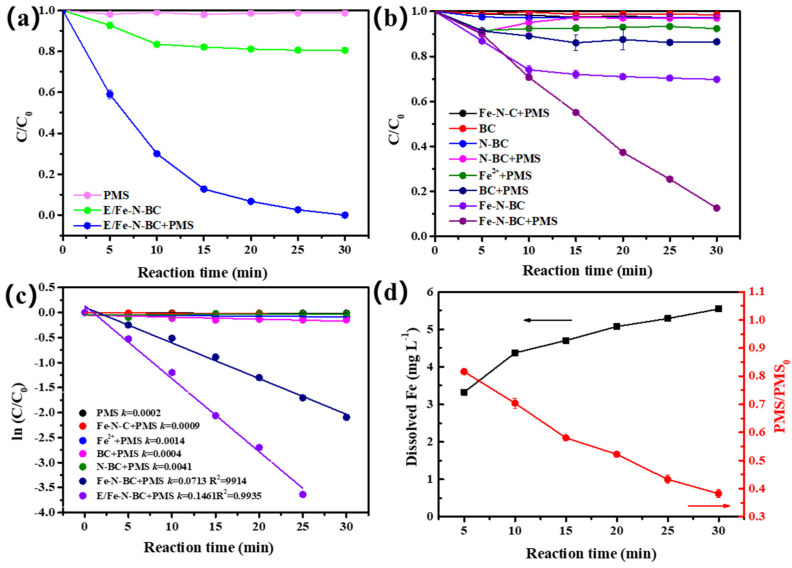
Removal efficiency of QNC in various systems (**a**,**b**). Reaction kinetics of QNC in different reaction systems (**c**). Residual rate of PMS and dissolved Fe in E/Fe–N–BC/PMS system (**d**). Reaction condition: [QNC] = 10 mg·L^−1^, pH= 4.2, T = 25 ± 1 °C, [catalyst] = 0.3 g·L^−1^, and [PMS] = 0.8 mM.

The catalytic performance of the obtained materials was evaluated by the removal of QNC within 30 min. QNC removal efficiency and rate was evaluated for all systems, including PMS alone and Fe (Ⅱ)/PMS (Figure 3a–c). Without PMS, the QNC was adsorbed by materials; compared with BC (1.51%) and N–BC (2.71%), the addition of iron enhanced the adsorption of QNC by Fe–N–BC (30.24%) and E/Fe–N–BC (19.46%), even though the specific surface area decreased. When PMS was added, the QNC was degraded. The weak degradation effect (1.23%, *k_obs_* = 0.0002 min^−1^) of QNC indicates that HSO_5_^−^ can decompose itself to produce SO_4_•^−^, according to Equation (3). Likewise, the homogeneous system of Fe^2+^/PMS (7.64%, *k_obs_* = 0.0014 min^−1^) shows poor removal efficiency for QNC because the homogeneous system easily produces sludge precipitation, resulting in rapid consumption of Fe^2+^. Regardless of whether PMS was added, the removal of QNC by BC and N–BC had no significant change, indicating that they had no catalytic effect on PMS. The degradation effect of Fe–N–C was only 3% (*k_obs_* = 0.0009 min^−1^), suggesting that the Fe–N–C had no catalytic activity on PMS. However, the degradation effect of Fe–N–BC/PMS (87.35%, *k_obs_* = 0.0713 min^−1^) increased sharply due to the formation of mixed–phase iron oxides (γ–Fe_2_O_3−x_) with high content Fe (II). When EDTA was added, the removal rate of E/Fe–N–BC (100%, *k_obs_* = 0.1461 min^−1^) further augmented, owing to the increased content of Fe^2+^ (Figure 2b). This is because the existence of Fe (II) is conducive to promoting the decomposition of PMS to produce SO_4_•^−^ and •OH, which accelerated the degradation of QNC. The resistivity is an important approach to evaluate the electron transfer ability of the material [43]. In comparison with Fe–N–BC, the addition of EDTA was conducive to reducing the resistance and accelerating the electron transfer rate of E/Fe–N–BC (Figure S8). In addition, after the addition of EDTA, the dispersion of spherical γ–Fe_2_O_3−x_ particles on the surface of biochar also increased (Figure 1d), which was beneficial to the enhancement of the contact area between the catalysts and PMS solution. These results all contributed to the excellent catalytic performance of E/Fe–N–BC. Furthermore, it was found that the iron dissolution and the residual rate of PMS in E/Fe–N–BC/PMS system were 5.544 mg/L and 38.28%, respectively (Figure 3d), indicating that this system had the advantages of low iron leaching and high utilization of PMS.

(3) $${\mathrm{HSO}}_{5}^{-}+e^{-}\to {\mathrm{SO}}_{4}{•}^{-}+{\mathrm{OH}}^{-}$$

#### 3.2.2. Effect of Catalyst Dosage on the Removal of QNC

**Figure 4:**
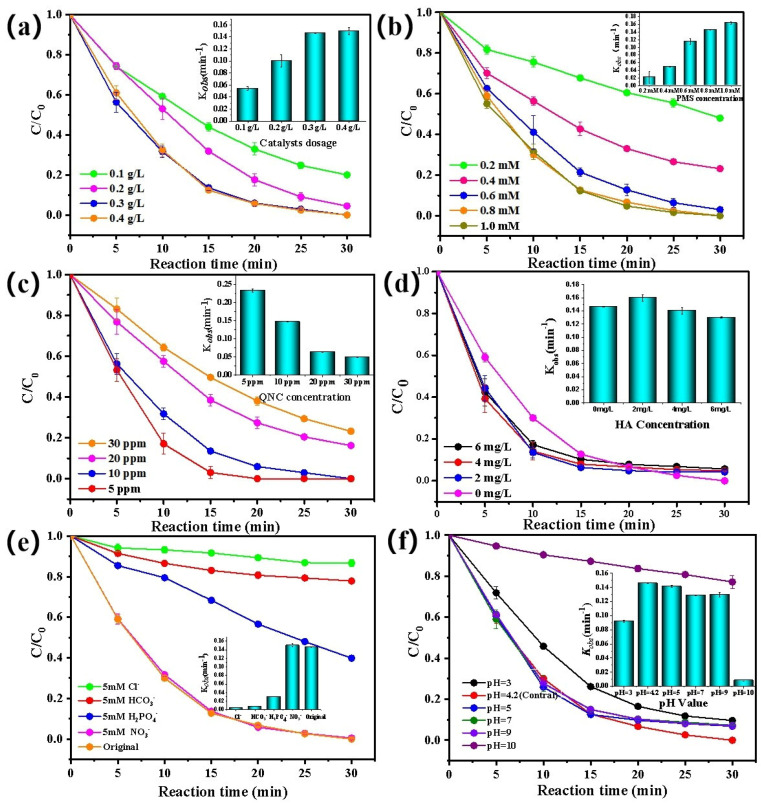
Effects of catalyst dosage (**a**), concentration of PMS (**b**), initial QNC concentration (**c**), HA (**d**), inorganic anions (**e**), and initial pH (**f**) in E/Fe–N–BC/PMS system. Reaction condition: [QNC] = 10 mg·L^−1^, [catalyst] = 0.3 g·L^−1^, [PMS = 0.8 mM], pH = 4.2, T = 25 ± 1 °C.

The effect of the dosage of E/Fe–N–BC catalyst on the degradation of QNC was investigated (Figure 4a). From Figure 3a, it should be noted that QNC cannot be effectively removed with PMS alone. Nevertheless, when the dosage of the catalyst was 0.1 g·L^−1^, the removal rate of QNC was 79.95% (*k_obs_* = 0.0543 min^−1^). With the increase of the catalyst from 0.1 g·L^−1^ to 0.3 g·L^−1^, the removal efficiency continuously augmented from 79.95% (*k_obs_* = 0.0543 min^−1^) to 100% (*k_obs_* = 0.1461 min^−1^) due to the increase of the active species in the reaction system with the enlargement of the catalytic dosage. However, when the catalyst was further magnified to 0.4 g·L^−1^, the removal rate (*k_obs_* = 0.1493 min^−1^) remained unchanged. This is because a massive catalyst activates PMS to produce excessive free radicals, which can cause a self–quenching effect, according to Equations (4) and (5). Therefore, the optimum catalyst dosage was 0.3 g·L^−1^.

(4) $${\mathrm{SO}}_{4}{•}^{-}+{\mathrm{SO}}_{4}{•}^{-}\to S_{2}O_{8}{}^{2-}$$

(5) $${\mathrm{SO}}_{4}{•}^{-}+•\mathrm{OH}\to {\mathrm{HSO}}_{5}{}^{-}$$

#### 3.2.3. Effect of PMS Concentration on the Removal of QNC

The effect of initial PMS concentration on QNC removal was also studied. As shown in Figure 4b, the increase of PMS dosage played a positive role in QNC degradation. When the initial PMS concentration increased from 0.2 mM to 0.8 mM, the removal rate of QNC enhanced from 51.96% (*k_obs_* = 0.022 min^−1^) to 100% (*k_obs_* = 0.1461 min^−1^). This is because increasingly more active species would be produced with the augmentation of PMS concentration, resulting in the enhancement of QNC removal. However, at the overdose of PMS from 0.8 mM to 1.0 mM, the removal efficiency remained invariable, and there was only a slightly enlarged reaction rate because the active component of the catalysts was consumed and/or occupied completely. Considering the economic and cost advantage, the initial PMS concentration was selected as 0.8 mM.

To further investigate the mineralization effect of the E/Fe–N–BC/PMS system, TOC tests were performed on the reaction system. From Figure S9, it can be seen that the TOC concentration of pure QNC was 5.60 mg/L. After only 0.8 mM PMS was added, the TOC concentration (5.69 mg/L) was close to pure QNC, indicating that the PMS–only solution could not degrade QNC. After only E/Fe–N–BC was added, the TOC increased substantially to 48.86 mg/L. This is because E/Fe–N–BC is a biochar–based catalysis, which is rich in soluble organic matter. Therefore, this phenomenon can be attributed to the leaching of soluble organic matter from E/Fe–N–BC into the reaction solution. Moreover, in the E/Fe–N–BC/PMS system, when the amount of PMS was 0.4 mM, the TOC of the reaction solution was 40.54 mg/L. When the dosage of PMS further enhanced to 0.8 mM, the TOC decreased to 33.54 mg/L, suggesting that the E/Fe–N–BC catalyst had an excellent catalytic effect on PMS for the mineralization of QNC. When the PMS further increased to 2.0 mM, the TOC (33.50 mg/L) was basically unchanged compared with 0.8 mM PMS. Additionally, as shown in Figure 4b, 0.4 mM PMS had an approximate 70% removal rate of QNC, and when the PMS dosage was higher than 0.8 mM, the QNC removal rate reached as high as 100%. It can be seen that the QNC removal rate had a similar variation tendency to TOC. This indicates that QNC may be mineralized into CO_2_ and H_2_O by the E/Fe–N–BC/PMS system, and the highest mineralization rate was found at 0.8 mM PMS.

#### 3.2.4. Effect of Initial QNC Concentration on the Removal of QNC

The removal rates of different initial concentrations of QNC were also investigated. As depicted in Figure 4c, it was clear that the degradation efficiencies and their rate constants of QNC slightly attenuated with the augmentation of their initial concentrations. After reaction 30 min, when the initial concentration of QNC was 5 mg·L^−1^, 10 mg·L^−1^, 20 mg·L^−1^, 30 mg·L^−1^, and 40 mg·L^−1^ the removal rates were 100%, 100%, 83.74%, 76.83%, and 69.86%, respectively. The corresponding reaction rates were 0.2326 min^−1^, 0.1416 min^−1^, 0.0631 min^−1^, 0.0488 min^−1^, and 0.0403 min^−1^. This phenomenon can be attributed to the fact that the active species were insufficient for the degradation of QNC due to the thorough consumption of PMS with the increase of their initial concentration. This also means that the E/Fe–N–BC/PMS system can have a good removal effect on high concentration of organic pollutants.

#### 3.2.5. Effect of HA and Anions on the Removal of QNC

Humic acid (HA), a ubiquitous natural organic substance in the environment, would generate competition with QNC during AOPs. Herein, the effects of HA concentrations at 2 mg·L^−1^, 4 mg·L^−1^, and 6 mg·L^−1^ on the removal of QNC were studied, as depicted in Figure 4d. Compared with the control experiment, the introduction of HA had little impact on QNC degradation, where the QNC removal efficiencies were 95.75% (*k_obs_* = 0.1597 min^−1^), 95.20% (*k_obs_* = 0.1302 min^−1^), and 94.28% (*k_obs_* = 0.1297 min^−1^) at initial HA concentrations of 2, 4, and 6 mg·L^−1^, respectively. This result indicates that E/Fe–N–BC showed excellent anti–interference capacity to natural organic substance.

Inorganic ions, such as Cl^−^, HCO_3_^−^, H_2_PO_4_^−^, and NO_3_^−^, are widely distributed in real aquatic systems. In AOPs, they have unavoidable interference effects on the decontamination of the organic pollutants. Therefore, the effects of Cl^−^, HCO_3_^−^, H_2_PO_4_^−^, and NO_3_^−^ (5 mM) on the removal rates of QNC were evaluated, as shown in Figure 4e. The results indicate that the influence of co–existing anions complied with the order of Cl^−^ > HCO_3_^−^ > H_2_PO_4_^−^ > NO_3_^−^. According to Equations (6) and (7), NO_3_^−^ could react with SO_4_•^−^ and •OH to generate NO_3_•. Although the oxidation potential of NO_3_• was slightly lower than that of SO_4_•^−^ (E^0^ = 2.5–3.1 V) and •OH (E^0^ = 2.8 V), NO_3_^−^ did not affect the QNC removal (99.52%, *k_obs_* =0.1501 min^−1^), while the other anions inhibited the degradation of QNC to some degree. The results showed that the degradation effect of QNC in the presence of Cl^−^, HCO_3_^−^, and H_2_PO_4_^−^ was 13.31% (*k_obs_* = 0.0047 min^−1^), 22.16% (*k_obs_* = 0.0.008 min^−1^), and 60.81% (*k_obs_* =0.0304 min^−1^), respectively. Previous reports have demonstrated that Cl^−^ could react with SO_4_•^−^, •OH, and PMS to produce low active Cl•, Cl_2_•^−^, Cl_2_, and HOCl species, according to Equations (9)–(14) [44]. Particularly, Cl^−^ was a quencher of SO_4_•^−^ and •OH, and this may have been the major factor for the decrease of QNC removal. The presence of HCO_3_^−^ and H_2_PO_4_^−^ could easily chelate on the surface of E/Fe–N–BC, which reduced the active sites of catalysts and hindered the formation of SO_4_•^−^ and •OH. Meanwhile, they would quench radicals to produce other low active species, such as HCO_3_• [23], reducing the degradation ability of QNC, respectively.

(6) $${\mathrm{NO}}_{3}{}^{-}+{\mathrm{SO}}_{4}{•}^{-}\to {\mathrm{NO}}_{3}•+{\mathrm{SO}}_{4}{}^{2-}\;(E^{0}\;({\mathrm{NO}}_{3}•)=2.3\;V)$$

(7) $${\mathrm{NO}}_{3}{}^{-}+•\mathrm{OH}\to {\mathrm{NO}}_{3}•+{\mathrm{OH}}^{-}$$

(8) $${\mathrm{NO}}_{3}•+H_{2}O+e_{\mathrm{aq}}\to {\mathrm{NO}}_{2}•+2{\mathrm{OH}}^{-}\;(E^{0}\;({\mathrm{NO}}_{2}•)=1.03\;V)$$

(9) $${\mathrm{Cl}}^{-}+{\mathrm{SO}}_{4}{•}^{-}\to \mathrm{Cl}•+{\mathrm{SO}}_{4}{}^{2-}\;(E^{0}\;(\mathrm{Cl}•)=2.4\;V)$$

(10) $${\mathrm{Cl}}^{-}+•\mathrm{OH}\;+{\;H}^{+}\;\to \;\mathrm{Cl}•+{\;H}_{2}O\;$$

(11) $${\mathrm{Cl}}^{-}+\mathrm{Cl}•\to {\mathrm{Cl}}_{2}{•}^{-}\;(E^{0}\;({\mathrm{Cl}}_{2}•{}^{-})=2.1\;V)$$

(12) $$2{\mathrm{Cl}}^{-}+2{\mathrm{HSO}}_{5}{}^{-}+2H^{+}\to 2{\mathrm{SO}}_{4}{}^{2-}+{\mathrm{Cl}}_{2}+2H_{2}O\;(E^{0}\;({\mathrm{Cl}}_{2})=1.4\;V)$$

(13) $${\mathrm{Cl}}_{2}+H_{2}O\to \mathrm{HOCl}\;(E^{0}\;(\mathrm{HOCl})=1.6\;V)$$

(14) $${\mathrm{Cl}}^{-}+{\mathrm{HSO}}_{5}{}^{-}\to {\mathrm{SO}}_{4}{}^{2-}+\mathrm{HOCl}$$

(15) $${\mathrm{HCO}}_{3}{}^{-}+{\mathrm{SO}}_{4}{•}^{-}\to {\mathrm{HCO}}_{3}•+{\mathrm{SO}}_{4}{}^{2-}\;(E^{0}\;({\mathrm{HCO}}_{3}•)=1.5\;V)$$

(16) $${\mathrm{HCO}}_{3}{}^{-}+•\mathrm{OH}\to {\mathrm{CO}}_{3}{}^{2-}+H_{2}O$$

(17) $$H_{2}{\mathrm{PO}}_{4}{}^{2-}+•\mathrm{OH}\to H_{2}{\mathrm{PO}}_{4}{•}^{-}+{\mathrm{OH}}^{-}$$

(18) $$H_{2}{\mathrm{PO}}_{4}{}^{2-}+{\mathrm{SO}}_{4}{•}^{-}\to H_{2}{\mathrm{PO}}_{4}{•}^{-}+{\mathrm{SO}}_{4}{}^{2-}\;$$

#### 3.2.6. Effect of pH on the Removal of QNC

Initial pH is a key factor affecting the catalytic performance in AOP systems [45]. Thus, the effects of pH on QNC removal were studied, as presented in Figure 4f. The initial solution pH is 4.24, in which the QNC were completely removed within 30 min. Since PMS is considered to be a buffer medium, the removal rate of QNC varied slightly with the removal efficiencies of 93.16% (*k_obs_* = 0.1415 min^−1^), 92.72% (*k_obs_* = 0.1288 min^−1^), and 92.9% (*k_obs_* = 0.1298 min^−1^) at initial pH of 5, 7, and 9, respectively. However, when pH was adjusted to excessive acidic and alkali, the removal efficiency of QNC was greatly affected, especially in the latter. They were drastically reduced to 90.36% (*k_obs_* = 0.0923 min^−1^) and 22.81% (*k_obs_* = 0.0084 min^−1^) at pH of 3 and 10, respectively. Hence, environmental pH has an important influence on the removal rate of QNC. This may have been caused by the activity of SO_4_•^−^ and •OH and the active site and surface charge of catalysts. Firstly, excess H^+^ can react with SO_4_•^−^ and •OH at pH 3, (Equations (19) and (20)) [25]. Similarly, at pH 10, excess OH^−^ can also react with SO_4_•^−^ (Equation (21)). Secondly, at pH 10, the surface Fe(II) irons of E/Fe–N–BC may be passivated to produce inactive FeOH^+^ and Fe(OH)_2_ [44]. As a result, the active sites were covered and would not contact with PMS to produce active species. Finally, as demonstrated in Figure S10, the surface charge of E/Fe–N–BC was electronegative at pH > 2.6, which increased gradually with the augmentation of solution pH. In addition, QNC would be dissociated into electronegative QNC^−^, according to its pKa of 4.34 [46]. Therefore, there was a strong repulsion between E/Fe–N–BC and QNC at pH 10, which further inhibited the degradation of QNC. Therefore, the E/Fe–N–BC/PMS systems are suitable for the removal of QNC under neutral and weak alkaline conditions.

(19) $${\mathrm{SO}}_{4}{•}^{-}+H^{+}+e_{\mathrm{aq}}{}^{-}\to {\mathrm{HSO}}_{4}{}^{-}$$

(20) $$•\mathrm{OH}+H^{+}+e_{\mathrm{aq}}{}^{-}\to H_{2}O$$

(21) $${\mathrm{SO}}_{4}{•}^{-}+{\mathrm{OH}}^{-}\to {\mathrm{SO}}_{4}{}^{2-}+•\mathrm{OH}$$

### 3.3. Radicals Identification

**Figure 5:**
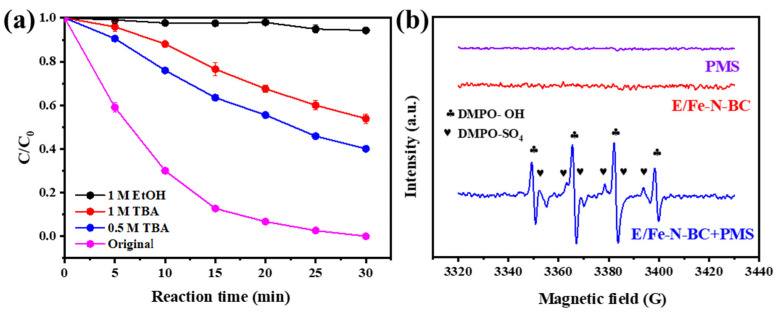
The effects of scavengers on the degradation of QNC in the E/Fe–N–BC/PMS system (**a**), EPR spectra of PMS, E/Fe–N–BC, and E/Fe–N–BC /PMS (**b**). Reaction condition: [QNC] = 10 mg/L, [catalyst] = 0.3 g/L, [PMS = 0.8 mM], pH = 4.2, T = 25 ± 1 °C.

In order to verify the active species in the degradation process, quenching experiments were carried out on the system. SO_4_•^−^ and •OH were identified by the EtOH and TBA [25]. Owing to the different quenching rates, EtOH is generally considered to be a scavenger of SO_4_•^−^ and •OH: (k_1_ (EtOH for SO_4_•^−^) = 1.6–7.7 × 10^7^ M^−1^s^−1^) and (k_2_ (EtOH for •OH) = 1.2–2.8 × 10^9^ M^−1^s^−1^) [47]. However, TBA had quenching effect only on •OH: (k_1_ (TBA for •OH)= (3.8–7.6) × 10^8^ M^−1^s^−1^) and (k_2_ (TBA for SO_4_•^−^) = (4–9.1) × 10^5^ M^−1^ s^−1^) [13]. Figure 5a and Figure S11 show the effect of EtOH and TBA quenching on the removal of QNC in the E/Fe–N–BC/PMS system. When 1 M EtOH was used as a quenching agent, the removal rate of QNC was only 5.7% (*k_obs_* = 0.0019 min^−1^). This result indicates that only SO_4_•^−^ and •OH existed in the system. In order to further analyze the free radical that played the dominant contribution to degradation reaction, 0.5 M and 1 M TBA were added to the system for •OH quenching. It was found that the removal rate of QNC decreased to 59.92% (*k_obs_* = 0.0316 min^−1^) and 46.11% (*k_obs_* = 0.0218 min^−1^), respectively, when the TBA concentration increased from 0.5 M to 1 M. According to the quenching results, both SO_4_•^−^ and •OH played a crucial role in the degradation of QNC. Moreover, the contribution of •OH to the degradation effect was slightly greater than that of SO_4_•^−^. 

The free radicals were further identified by EPR (electron paramagnetic resonance) analysis vis using DMPO (5,5–dimethyl–1–pyrroline N–oxide) as a trapping agent. In general, metal catalysts can activate PMS to produce free radicals [17]. From Figure 5b, when either PMS or E/Fe–N–BC existed alone, no signals were observed. On the contrary, obvious EPR signals with peak intensity ratios of 1:2:2:1 in the E/Fe–N–BC/PMS system appeared [48]. This is a typical signal for DMPO–OH, suggesting the formation of hydroxyl radicals (•OH). Moreover, weak DMPO–SO_4_ signals were also produced in this system, indicating the presence of SO_4_•^−^ [49]. This result further confirmed that both SO_4_•^−^ and •OH were the primary active species in the E/Fe–N–BC/PMS system, which is consistent with the above quenching analyses. 

### 3.4. Possible Degradation Mechanisms

**Figure 6:**
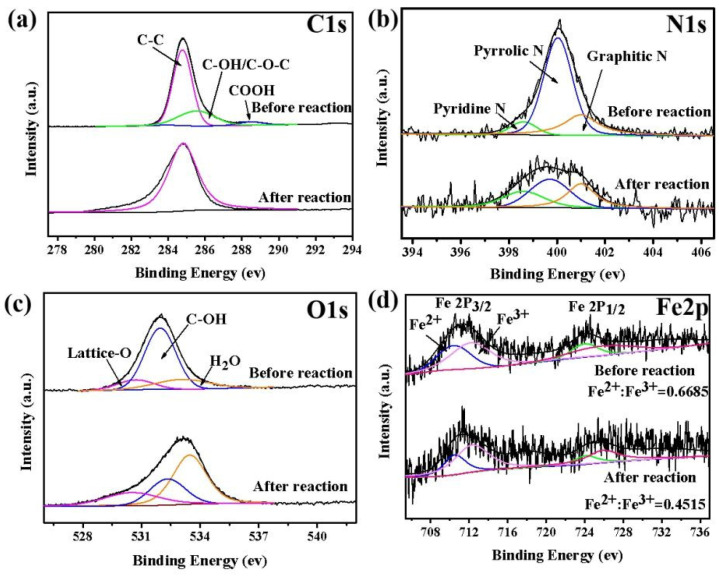
The XPS spectra of C 1s (**a**), N 1s (**b**), O 1s (**c**), and Fe 2p (**d**) before and after reaction in the E/Fe–N–BC/PMS system.((**a**) pink line: C–C, cyan line: C–OH/C–O–C, blue line: –COOH. (**b**) orange line: graphitic N, cyan line: pyridine N, blue line: pyrrolic N. (**c**) red line: lattice O, cyan line: C–OH, blue line: H_2_O. (**d**) pink and cyan line: Fe^2+^; blue and red line: Fe^3+^).

To identify the possible degradation mechanisms, the XPS spectra of the E/Fe–N–BC catalyst before and after reaction were investigated. Before reaction, the C 1s structure (Figure 6a) could be fitted to three components at 284.78 eV (C–C), 285.56 eV (C–O–C/C–OH), and 288.48 eV (COOH), with the corresponding proportions of 69.81%, 4.08%, and 26.11%, respectively [27]. However, only the C–C (284.8 eV) peak existed on the C structure after reaction. This may be caused by the dissolution of the substance with hydrophilic groups. The N 1s spectra before reaction were fitted in Figure 6b, where three peaks at ~398.6 eV, ~400 eV, and ~401 eV corresponded to pyridinic–N (7.64%), pyrrolic–N (68.57%), and graphitic–N (23.79%), respectively [50]. It has been reported that pyridine N and graphite–N can improve the interface affinity of carbon scaffolders, and graphite–N also plays an important role in improving the catalytic activity of N–doped carbon materials in AOPs [51]. The O 1s spectra before reaction were fitted in Figure 6c, where three peaks at 530.5 eV, 532.2 eV, and 533.3 eV corresponded to lattice–O (11.95%), C–OH (68.18%), and surface–bound H_2_O (19.87%), respectively [37]. After reaction, the significant reduction of C–OH was beneficial for accelerating the decomposition of PMS. More importantly, the high–resolution Fe 2P spectrum showed that the proportion of Fe^2+^/Fe^3+^ decreased from 0.6685 to 0.4515, which was accompanied by an Fe 2p_3/2_ shift to higher binding energies [52]. This indicates that Fe (Ⅱ) participated in the activation of PMS in the reaction process [53].

**Figure 7:**
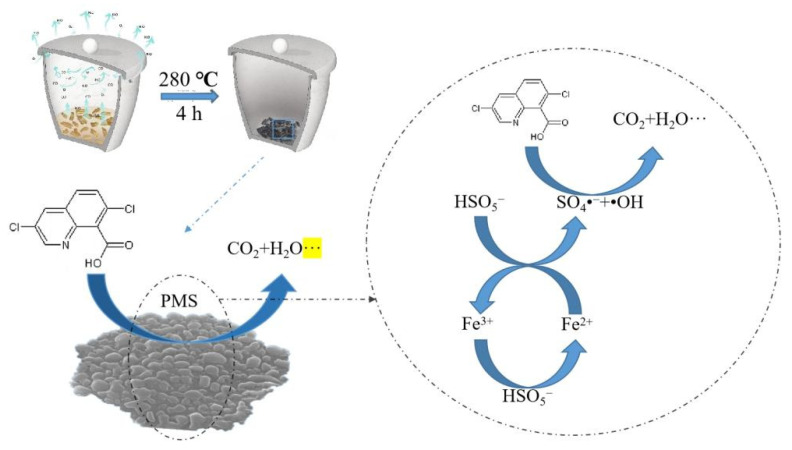
The possible degradation mechanisms of QNC in the E/Fe–N–BC/PMS system.

From the above radical quenching and XPS analysis results, a possible mechanism of PMS activation was proposed. The high content Fe (II) in mixed–phase γ–Fe_2_O_3−x_ had an important part in the activation of PMS, as shown in Equation (2). During the reaction process, SO_4_•^−^ would react with H_2_O/OH^−^ to produce •OH (Equations (22) and (23)). As a result, QNC was attacked by SO_4_•^−^ and •OH to form some intermediates (Figure S12), eventually producing CO_2_ and H_2_O (Equations (24) and (25)) [46,54]. The possible degradation mechanisms of QNC in the E/Fe–N–BC/PMS system was deduced and is described in Figure 7.

(22) $${\mathrm{SO}}_{4}{•}^{-}+{\mathrm{OH}}^{-}\to {\mathrm{SO}}_{4}^{2-}+•\mathrm{OH}$$

(23) $${\mathrm{SO}}_{4}{•}^{-}+H_{2}O\to {\mathrm{SO}}_{4}^{2-}+•\mathrm{OH}+H^{+}$$

(24) $${\mathrm{SO}}_{4}{•}^{-}+\mathrm{QNC}\to \mathrm{intermediates}\to {\mathrm{CO}}_{2}+H_{2}O\cdot \cdot \cdot$$

(25) $$•\mathrm{OH}+\mathrm{QNC}\to \mathrm{intermediates}\to {\mathrm{CO}}_{2}+H_{2}O\cdot \cdot \cdot$$

### 3.5. Stability and Practical Application of E/Fe–N–BC

The stability of the catalyst is also crucial for its practical application. The cyclic performance of E/Fe–N–BC was evaluated according to the method shown in Text S6, and the result is described in Figure S13. The catalytic activity of E/Fe–N–BC catalysts greatly receded after the third cycle, in which the removal rate of QNC decreased from 100% to 37.14% to 17.17% for the consecutive three times it was used. According to the above, the inactive reason might be attributed to the active substance iron leaching and the oxidation of Fe (II) on the surface of catalyst. 

**Figure 8:**
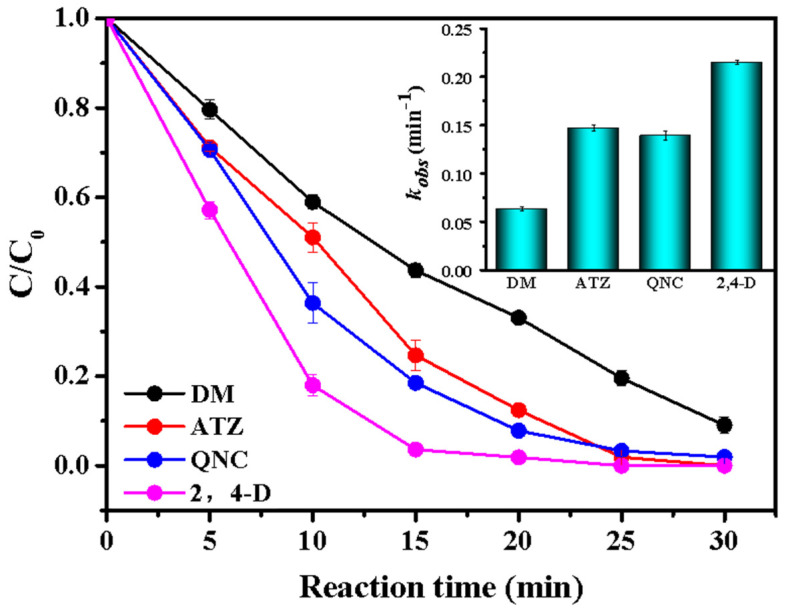
The application of schematic diagram of mass production of E/Fe–N–BC catalyst for the removal of different organic pollutions in the presence of PMS. Reaction condition: [catalyst] = 0.3 g/L, [PMS = 0.8 mM], T = 25 ± 1 °C, [DM] = [ATZ] = [QNC] = [2,4–D] = 10 mg/L.

The scaled–up production of the catalyst is a critical parameter for its practical application. Mass preparation of catalysts was carried out according to the method shown in Text S4 and Figure S14. To verify the catalytic properties, four different herbicides, including dicamba (DM), atrazine (ATZ), 2,4–dichlorophenoxyacetic acid (2,4–D), and QNC, were degraded by the material prepared from this method, and their removal efficiencies are described in Figure 8. It can be seen that the degradation effects of the four herbicides were excellent. Among them, 2, 4–D was completely removed after 25 min with a high reaction rate (*k_obs_* = 0.2153 min^−1^). The removal rate of DM, ATZ, and QNC reached 90.94% (*k_obs_* = 0.0.06345 min^−1^), 100% (*k_obs_* = 0.1471 min^−1^), and 98.05% (*k_obs_* = 0.1395 min^−1^), respectively. This means that the material prepared by mass production also has great potential for the removal of various organic pollutants.

## 4. Conclusions

In summary, γ–Fe_2_O_3−x_@biochar (E/Fe–N–BC) with high Fe (II) content was successfully prepared by a sol–gel method combined with low–temperature reduction. During the synthesis process, the sesame shell biomass was simultaneously used as carbon resource and reductant, while NaFeEDTA and EDTA were employed as an iron resource and complexing agent, respectively. The prepared E/Fe–N–BC possesses excellent catalytic performance to peroxymonosulfate (PMS) for the removal of QNC. The removal efficiency of 10 mg/L of QNC was 98.05% after 25 min using 0.3 g/L E/Fe–N–BC catalyst and 0.8 mM PMS. SO_4_•^−^ and •OH were the main active species in the oxidation process. The high content Fe (II) in E/Fe–N–BC material had an important part in the activation of PMS. This material also possesses the advantage of large–scale preparation. These findings provide a facile, rapid, and easy–to–operate strategy for the synthesis of high–performance mixed–phase iron oxides catalysts which can be used for the removal of organic pollutants in AOPs.

## Data Availability

A full questionnaire in Chinese may be provided upon request.

## Supplementary Materials

The following supporting information can be downloaded at: https://www.mdpi.com/article/10.3390/ijerph192416790/s1, Text S1: Gas collection experiments; Text S2: PMS residue concentration determination; Text S3: Electrochemical impedance spectroscopy (EIS) measurement; Text S4: Calculation of reaction rate; Text S5: Mass production technique; Text S6: Recycle and re–utilization of E/Fe–N–BC; Table S1: HPLC (Agilent 1260 Infinity II) conditions of different organic pollutants; Table S2: The element content of C, O, N and Fe obtained by XPS analysis; Table S3: Specific surface area, average pore size and average pore volume of BC, N–BC, Fe–N–BC, E/Fe–N–BC and Fe–N–C; Table S4: Abbreviations used in this paper; Figure S1: The SEM image of BC (a,b) and N–BC (c); Figure S2: The statistical analysis diagrams of particle sizes in E/Fe–N–BC and Fe–N–BC; Figure S3: The survey spectrum XPS of E/Fe–N–BC and Fe–N–BC; Figure S4: The XPS spectra of Fe 2P of Fe–N–C; Figure S5: The magnetic hysteresis loop of Fe–N–C, Fe–N–BC and E/Fe–N–BC; Figure S6: The TEM image of E/Fe–N–BC; Figure S7: the adsorption/desorption isotherms of Fe–N–C, BC, N–BC, Fe–N–BC and E/Fe–N–BC; Figure S8: The electrochemical impedance spectrum (EIS) of Fe–N–BC and E/Fe–N–BC; Figure S9: TOC concentration of different reagents after reacting with QNC for 30min; Figure S10: The zeta potential of E/Fe–N–BC at different pH; Figure S11: Reaction rates of QNC in the E/Fe–N–BC/PMS system with the addition of different scavenger; Figure S12: possible degradation intermediates of QNC in E/Fe–N–BC/PMS system; Figure S13: The reuse capacity of E/Fe–N–BC for QNC removal; Figure S14: Schematic diagram of material process for mass production. Reference [55] is cited in the supplementary materials.
