# Supplementary material for: Low-Temperature Reduction Synthesis of γ–Fe2O3−x@biochar Catalysts and Their Combining with Peroxymonosulfate for Quinclorac Degradation
Source: Int J Environ Res Public Health. 2022 Dec 14;19(24):16790. doi: 10.3390/ijerph192416790 (PMC9779240; doi:10.3390/ijerph192416790)
Supplement: Supplementary file 1 [file ijerph-19-16790-s001.zip › ijerph-2027865-supplementary.pdf]

Supporting Information

Low-Temperature Reduction Synthesis of γ–Fe_2O_{3-x}@biochar Catalysts and Their Combining with Peroxymonosulfate for Quinclorac Degradation

Mei-e Zhong [1,†], Gongsong Tong [1,†], Austin Merchant [2], Jingchun Sun [1], Nan Zhou [1], Chunxia Ding [1], Xuguo Zhou [2,*] and Xiangying Liu [2,3,4,*]

[1] School of Chemistry and Materials Science, Hunan Agricultural University, No.1 Nongda Road, Furong District, Changsha 410128, China
[2] Department of Entomology, University of Kentucky, S-225 Agricultural Science Center North, Lexington, KY 40546-0091, USA
[3] College of Plant Protection, Hunan Agricultural University, No.1 Nongda Road, Furong District, Changsha 410128, China
[4] Hunan Provincial Key Laboratory for Biology and Control of Weeds, Hunan Agricultural University, No.1 Nongda Road, Furong District, Changsha 410125, China
***** Correspondence: xuguozhou@uky.edu (X.Z.); lxy525525@163.com (X.L.)

† These authors contributed equally to this work.

Text S1. Gas collection experiments

In order to facilitate gas collection, the experiment was carried out in a tube furnace [1]. The raw materials were expanded by ten times according to the experiment processes shown in manuscript. Firstly, the materials were dispersed in a beaker with 30 mL. After dehydrated drying overnight, the mixture was transferred to a corundum crucible and placed in a quartz tube (D=50 mm, L=500 mm) with 2 mL/min N_2 as the carrier gas, followed by pyrolyzing at 280℃ with a heating rate of 10℃ min^{-1}. During the reaction, gas products were collected in 1 L gas-bags after thorough drying with silica-gel, and then analyzed using a GC instrument (GC-5890). The gases are taken during 45 min after reaction for 30 min.

Text S2. PMS residue concentration determination

After the oxidation by HSO_5^-, iodide ions were reduced into elemental iodine (Eq. 1), and then tested by UV spectrophotometer (UVMini-1280) at 352nm. Typically, 1 ml of the reaction solution was taken with a polyethylene needle and filtrated with 0.45 um membrane, The filtrate was added into 8 mL potassium iodide solution with a given concentration (10mM) (in order to prevent oxidation, a certain amount of sodium bicarbonate was previously added into), and then tested after reaction for 10 min.

Text S3. Electrochemical impedance spectroscopy (EIS) measurement

Impedance measurements were performed on an electrochemical workstation (Autolab83943, Metrohm) with three-electrode cell system (working electrode (glassy carbon electrode), counter electrode (platinum wire electrode) and reference electrode (Ag/AgCl)). The electrolyte was 1 M Potassium ferricyanide/potassium ferrocyanide. Electrochemical impedance spectroscopy (EIS) was measured frequency ranging from 0.1 Hz to 1MHz under the open-circuit potential (OCP).

Text S4. Calculation of reaction rate

The reaction rate was evaluated by pseudo-first order kinetic model as the following equation:

$$\ln\left(\frac{C_t}{C_0}\right) = -k_{obs} \cdot t$$

C_0: the initial pollutant concentration;

C_t: the concentration at a certain time t during the degradation process;

k_{obs}: the reaction rate constant.

Text S5. Mass production technique

3.5 g FeNaEDTA and 1.5 g EDTA dissolved in a 300 mL ceramic crucible containing 200 mL water. 10 g sesame shell was added after the solid is completely dissolved. Materials were prepared by the same preparation method as shown in the 2.2 Preparation of catalysts of the manuscript. Actual preparation picture was shown in Fig. S13.

Text S6. Recycle and re-utilization of E/Fe-N-BC

To examine the stability, catalysts were collected by filtering with a 100-mesh sieve, and then 100 mL QNC (10 mg/L) solution and 0.8 mL PMS (100 mM) solution were added to continue the reaction.

Table. S1. HPLC (Agilent 1260 Infinity II) conditions of different organic pollutants

Sample	Mobile phase (V/V)	Flaw rate （ml/min）	Column temperature (℃)	Sample injection volume (μL)	UV detection wavelength (nm)
Quinclorac	acetum (1 wt%)/methanol =40/60	1	30	20	240
2, 4-Dichlorophenoxyacetic acid	Phosphoric acid (1 w%）/acetonitrile=50/50	1	30	20	205
Atrazine	water/ methanol=40/60	1	30	20	230
Dicamba	Phosphoric acid (0.125 w. t%）- monopotassium phosphate (4 mM) /acetonitrile=50/50	1	30	20	210

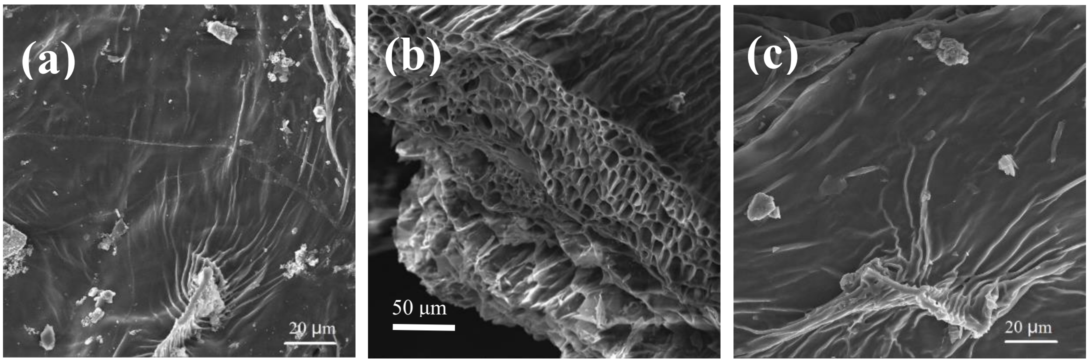

Figure S1 The SEM image of BC (a,b) and N-BC(c).

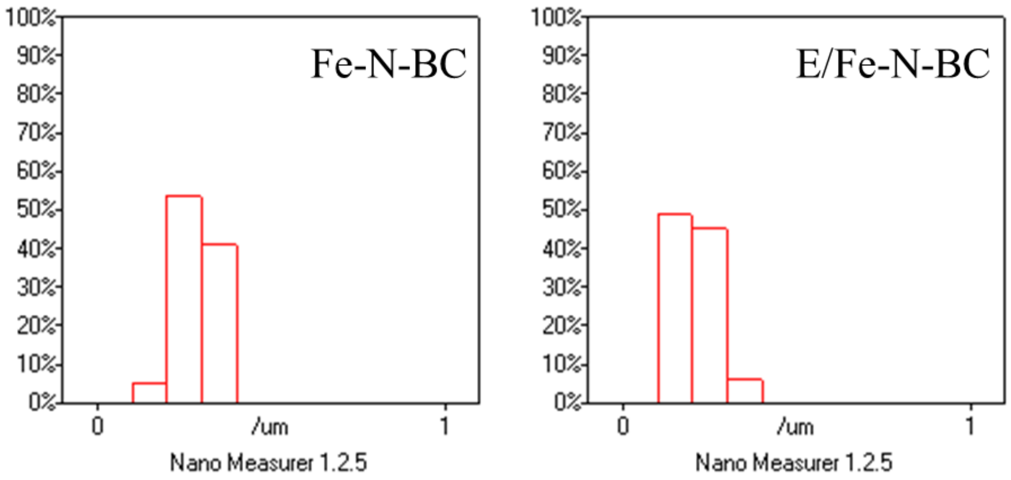

Figure S2 The statistical analysis diagrams of particle sizes in E/Fe-N-BC and Fe-N-BC

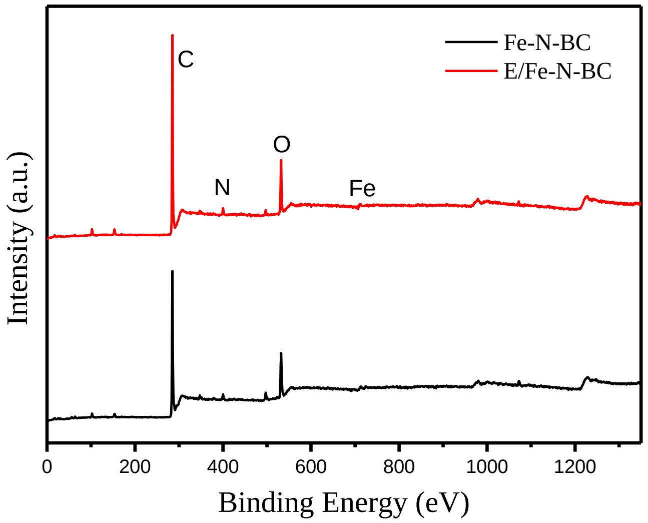

Figure S3 The survey spectrum XPS of E/Fe-N-BC and Fe-N-BC

Table S2 The element content of C, O, N and Fe obtained by XPS analysis.

Sample	C	O	N	Fe
Fe-N-BC	83.74%	13.09%	2.61%	0.56%
E/Fe-N-BC	85.37%	11.72%	2.46%	0.45%

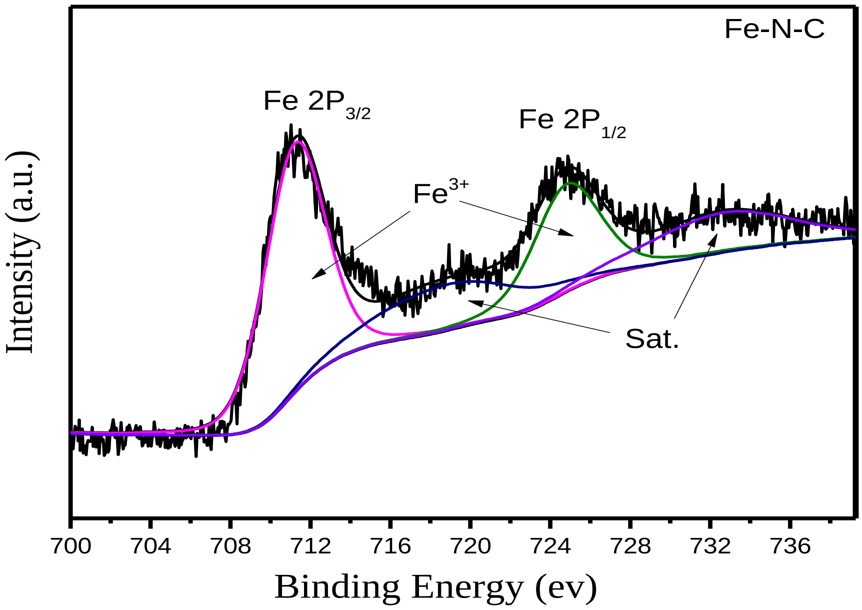

Figure S4 The XPS spectra of Fe 2P of Fe-N-C

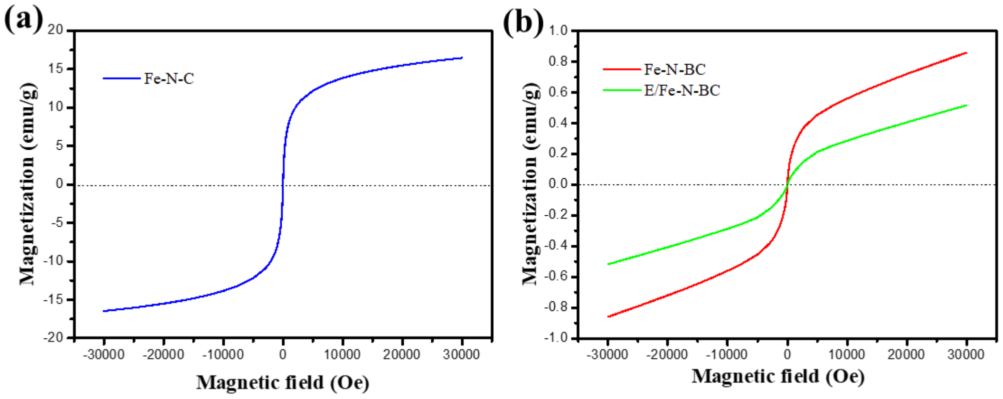

Figure S5 The magnetic hysteresis loop of Fe-N-C, Fe-N-BC and E/Fe-N-BC

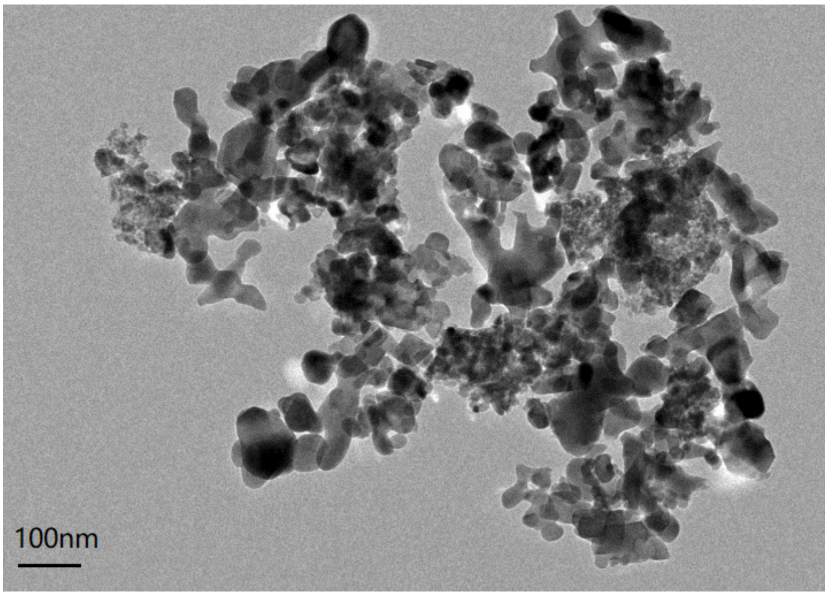

Figure S6 The TEM image of E/Fe-N-BC

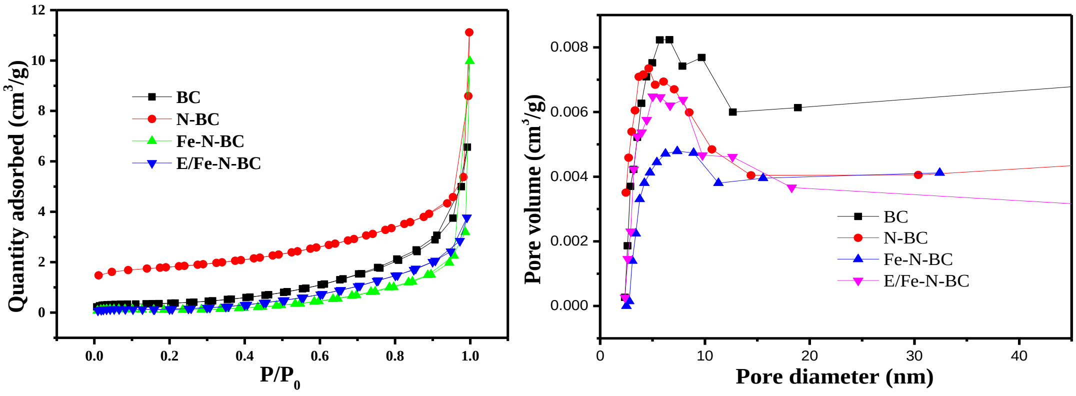

Figure S7 the adsorption/desorption isotherms of Fe-N-C, BC, N-BC, Fe-N-BC and E/Fe-N-BC

Table S3 Specific surface area, average pore size and average pore volume of BC, N-BC, Fe-N-BC, E/Fe-N-BC and Fe-N-C.

Sample	Specific surface area (m^2/g)	average pore size (nm)	average pore volume (cm^3/g)
BC	3.6458	6.23	0.01135
N-BC	7.5017	9.06	0.01600
Fe-N-BC	0.6202	16.88	0.01623
E/Fe-N-BC	0.6211	5.01	0.006171

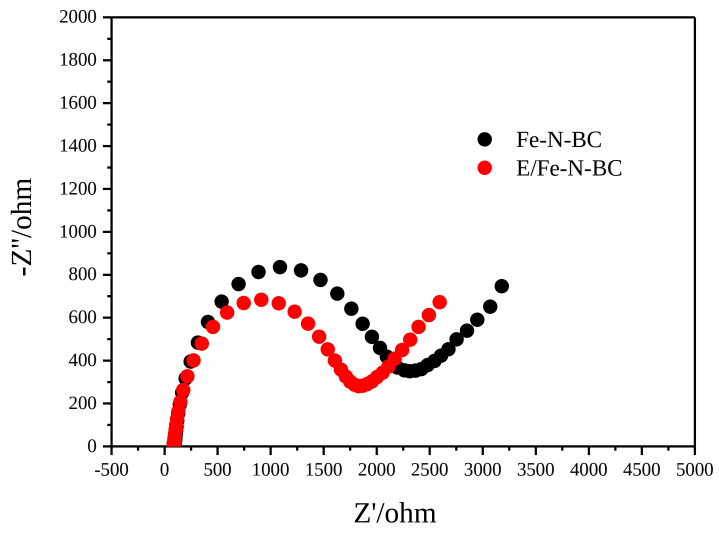

Figure S8 The electrochemical impedance spectrum (EIS) of Fe-N-BC and E/Fe-N-BC

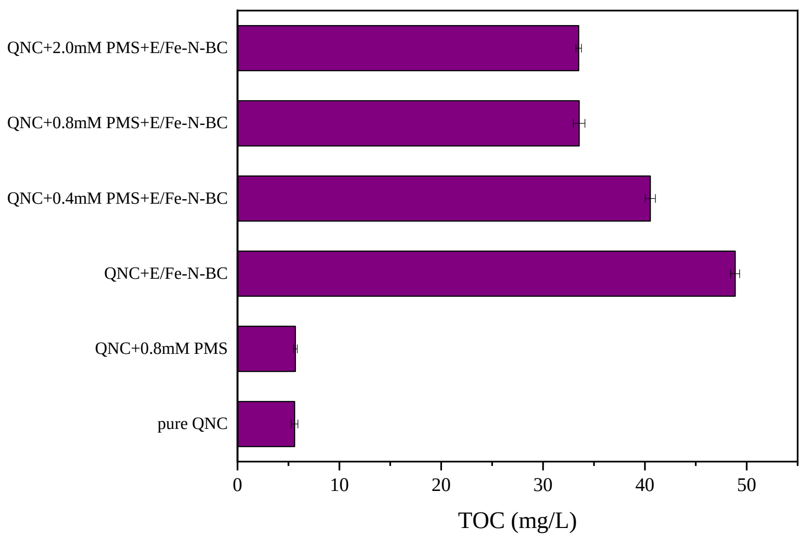

Figure S9 TOC concentration of different reagents after reacting with QNC for 30min.Reaction condition: [QNC]=10 $mg \cdot L^{-1}$, [catalyst]=0.3 $g \cdot L^{-1}$, pH=4.2, T=25±1 °C.

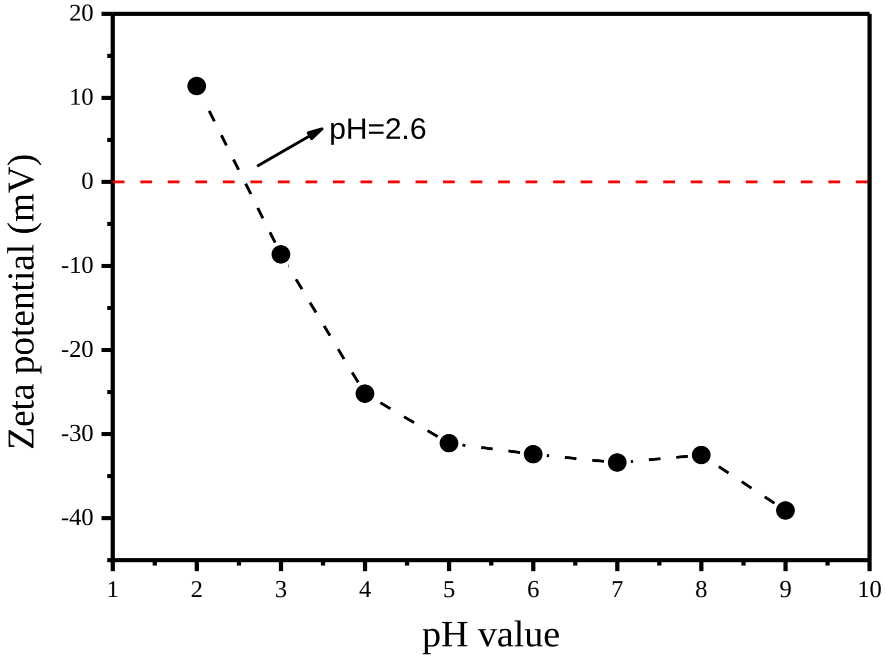

Figure S10 The zeta potential of E/Fe-N-BC at different pH

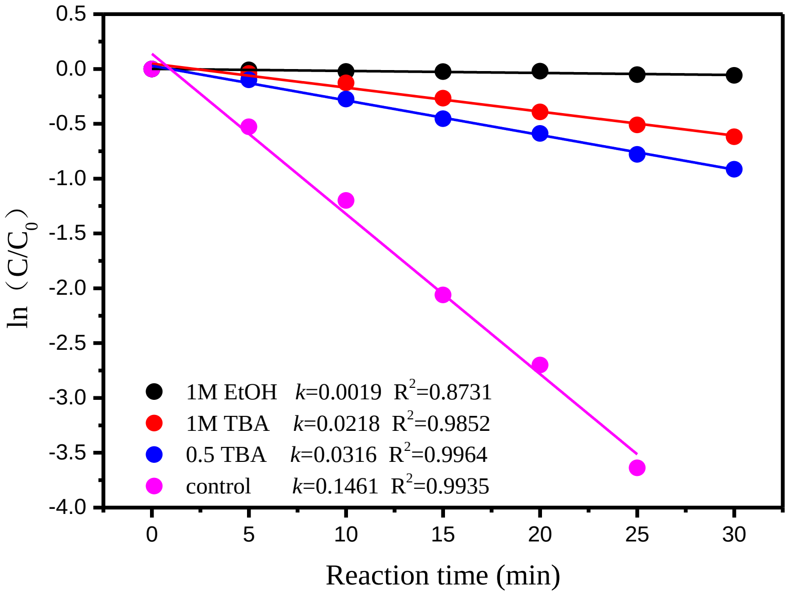

Figure S11 Reaction rates of QNC in the E/Fe-N-BC/PMS system with the addition of different scavenger

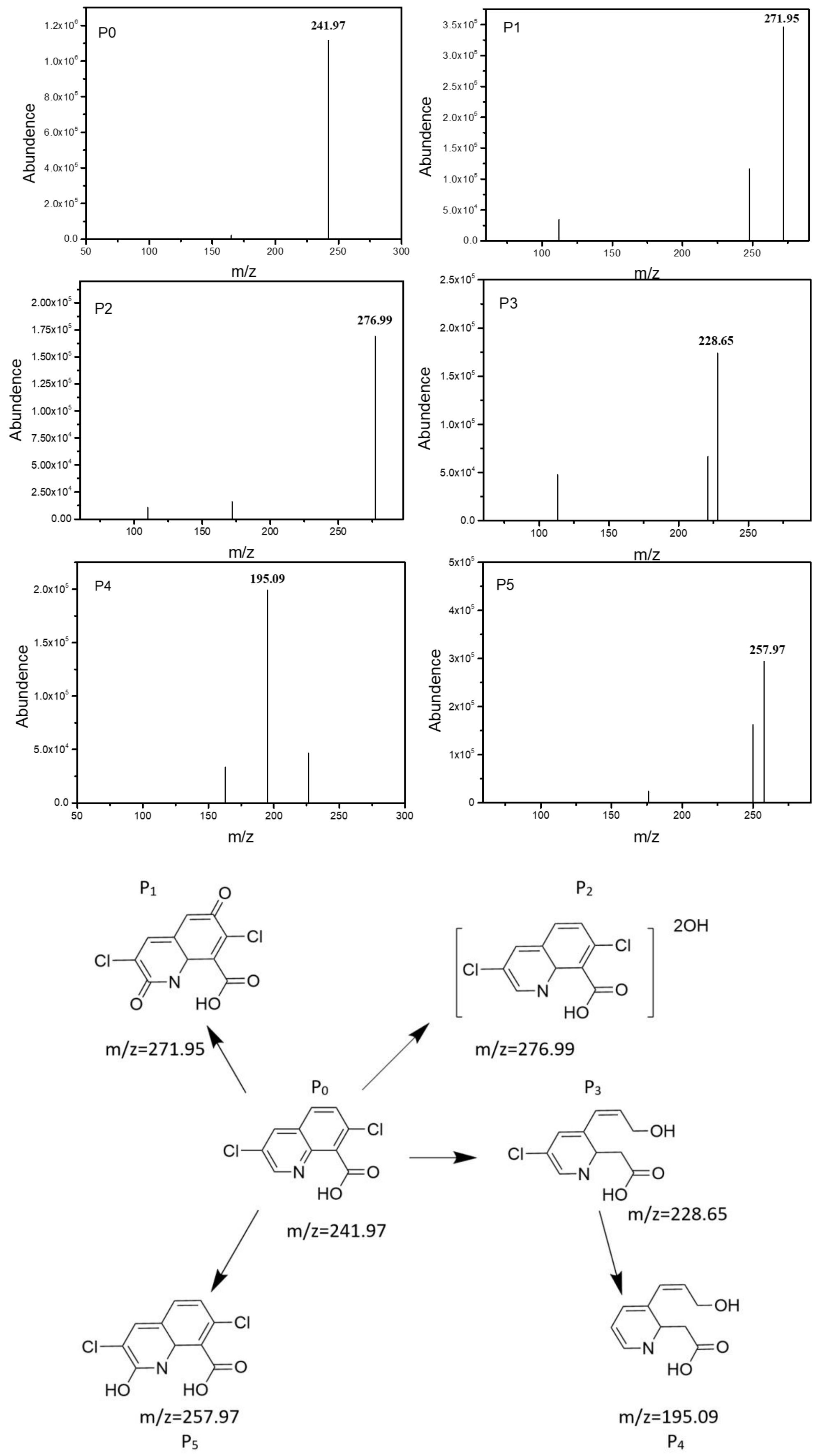

Figure S12 possible degradation intermediates of QNC in E/Fe-N-BC/PMS system

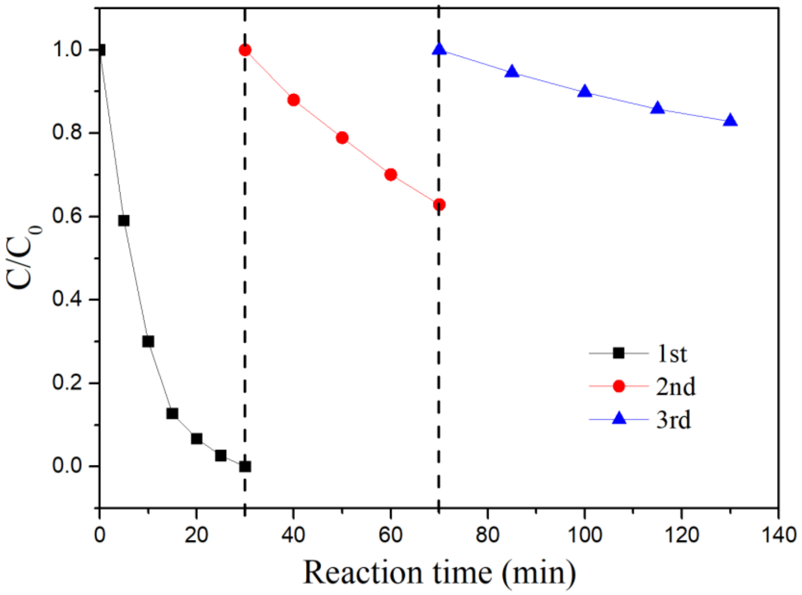

Figure S13 The reuse capacity of E/Fe-N-BC for QNC removal. [catalyst]=0.3 g·L^{-1}, [QNC]==10 mg·L^{-1}, [PMS]=0.8 mM, T=25°C±1.

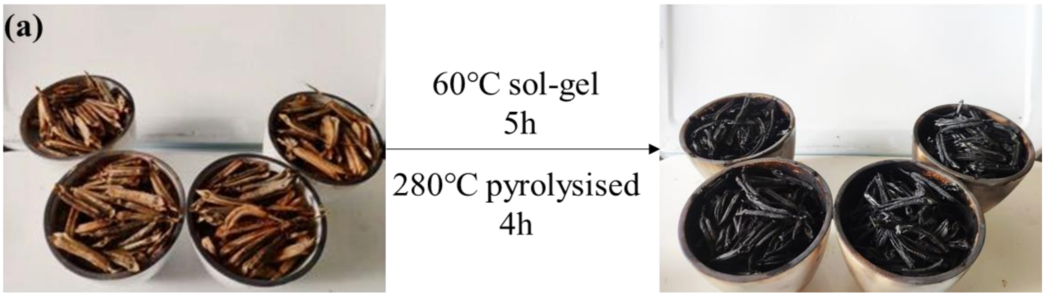

Figure S14 Schematic diagram of material process for mass production

Table. S4. Abbreviations used in this paper.

Abbreviations	Full name	Abbreviations	Full Name
E/Fe-N-BC	γ-Fe_2O_{3-x}@biochar	Fe-N-BC	E/Fe-N-BC without EDTA
N-BC	E/Fe-N-BC without FeNaEDTA	BC	E/Fe-N-BC without EDTA and FeNaEDTA
QNC	Quinclorac	PMS	Peroxymonosulfate
AOPs	Advanced oxidation processes	PDS	Peroxydisulfate
ROS	Reactive oxygen species	$SO_4\bullet^-$	Sulfate radicals
FeNaEDTA	Ethylenediamine tetraacetic acid ferric sodium salt	•OH	Hydroxyl radicals
EDTA	Ethylene diamine tetraacetic acid	HA	Humic acid
DM	Dicamba	2,4-D	2,4-dichlorophenoxyacetic acid
ATZ	Atrazine	EtOH	Ethanol
TBA	Tert butyl alcohol	XRD	X-ray diffraction
SEM	Scanning electron microscopy	TEM	Transmission electron microscopy
XPS	X-ray photoelectron spectroscopy	EDS	Energy-dispersive X-ray spectroscopy
VSM	Vibrating sample magnetometer	BET	Brunauer, Emmett and Teller specific surface area test method
FT-IR	Flourier transform infrared	EPR	Electron paramagnetic resonance
DMPO	5,5-dimethyl-1-pyrroline N-oxide	EIS	Electrochemical impedance spectroscopy
HPLC	High Performance Liquid Chromatography	UV	Ultraviolet

References for Supporting Information

[1] S. Huang, Y. Su, W. Luo, Q. He, S. Huang, N. Zhou, Z. Zhou, Kinetic analysis and in-situ no support catalytic pyrolysis product distribution of Chinese herb residue, Journal of Analytical and Applied Pyrolysis, 156 (2021) 105114.
